# Creating “boots on the ground”: addressing the shortage of field epidemiologists in the Philippines through intermediate-level training programmes

**DOI:** 10.5365/wpsar.2023.14.3.1053

**Published:** 2023-09-30

**Authors:** Rio Lat Magpantay, Ray Justin Cacho, Ventura, Mariz Zheila C Blanco, Apple Charm Agulto, Karen B Lonogan, Rosario P Pamintuan, Charmaine Madria-Barangan

**Affiliations:** a Department of Health, Manila, Philippines.

## Abstract

**Problem:**

As of 2022, only 49 graduates of the Philippines’ Field Epidemiology Training Programme (FETP) were employed by the Philippine Government, emphasizing the urgent need to increase the number of practicing field epidemiologists to better equip the country for public health emergencies.

**Context:**

The FETP–Intermediate Course (IC) curriculum is based mainly on the module of the United States Centers for Disease Control and Prevention that was incorporated into the Philippine context. It consists of five 1–2-week lecture series that provide participants with the knowledge and tools necessary to conduct job-relevant field projects. Individual projects are the centrepiece of the FETP–IC, requiring trainees to investigate outbreaks, design and develop protocols, conduct field data collection, manage data, analyse data, interpret data, write reports and deliver oral presentations.

**Action:**

To address the shortage of practicing field epidemiologists in the Philippines, a subnational initiative in Northern Luzon was implemented.

**Outcome:**

Within 3 years, the two FETP–IC subnational training programmes have produced 42 applied epidemiologists who will strengthen epidemiology and surveillance in their respective localities. As of February 2023, 92 studies have been conducted, including 39 outbreak investigations, 37 data quality analysis/process improvement projects, 10 epidemiological studies and six surveillance evaluations.

**Discussion:**

By training and deploying skilled epidemiologists to local health offices and hospitals, the programme is helping to improve the capacity of the health system to respond to public health threats and protect the health of the population. The programme’s emphasis on practical training and real-world experience is an effective way to build a strong and sustainable epidemiological workforce.

## PROBLEM

The Philippines’ 2-year Field Epidemiology Training Programme (FETP) was established in 1987 to train professional epidemiologists and develop a self-sustaining capacity for this training within the Department of Health (DOH). Over 35 years, the programme has produced 128 graduates (an average of three graduates per year). However, due to various circumstances, such as retirement, death and transfer to other positions, only 49 FETP graduates are currently employed in government service in the field of epidemiology. Among these, the distribution of epidemiologists is uneven, with only 22 graduates employed in local government units, 11 in the DOH Center for Health Development (CHD), seven in the DOH’s Central Office and Epidemiology Bureau, and six in hospitals, while the remaining three are employed in other government agencies. Moreover, the median age of practicing field epidemiologists in the country is 52, with 17 expected to retire in the next 5 years.

In a 2017 assessment carried out by the Training Program in Epidemiology and Public Health Interventions Network (TEPHINET), evaluators identified the need to expand the programme. The Joint External Evaluation of the implementation of the International Health Regulations (IHR 2005) in the Philippines in 2018 also recommended articulating three levels of FETP and ensuring adequate numbers are trained and available at the local, regional and national levels. Therefore, there was an urgent need to take proactive steps to increase the number of practicing field epidemiologists in the Philippines to ensure that the country is better equipped to handle public health emergencies in the future.

## CONTEXT

The FETP is a specialized training programme aimed at enhancing the skills of public health professionals, specifically epidemiologists, to respond effectively to public health threats. The programme offers hands-on training in applied epidemiology, outbreak investigation, surveillance and programme evaluation. Originally established in 1975 by the United States Centers for Disease Control and Prevention (CDC), the FETP has been adopted by many countries worldwide, including the Philippines, to develop a cadre of field epidemiologists who serve as a key component of the country’s public health system. (*1*) The FETP has been credited with facilitating early detection and response to outbreaks of infectious and noncommunicable diseases such as Ebola, SARS and COVID-19.

Globally, the FETP has evolved into a tiered programme, with an intermediate level added between the front-line and advanced levels, providing more structured career progression for field epidemiologists. (*2*) The intermediate level offers specialized training in topics such as outbreak investigation, data management and surveillance system design, building upon the core competencies learned at the front-line level.

**Fig. 1:**
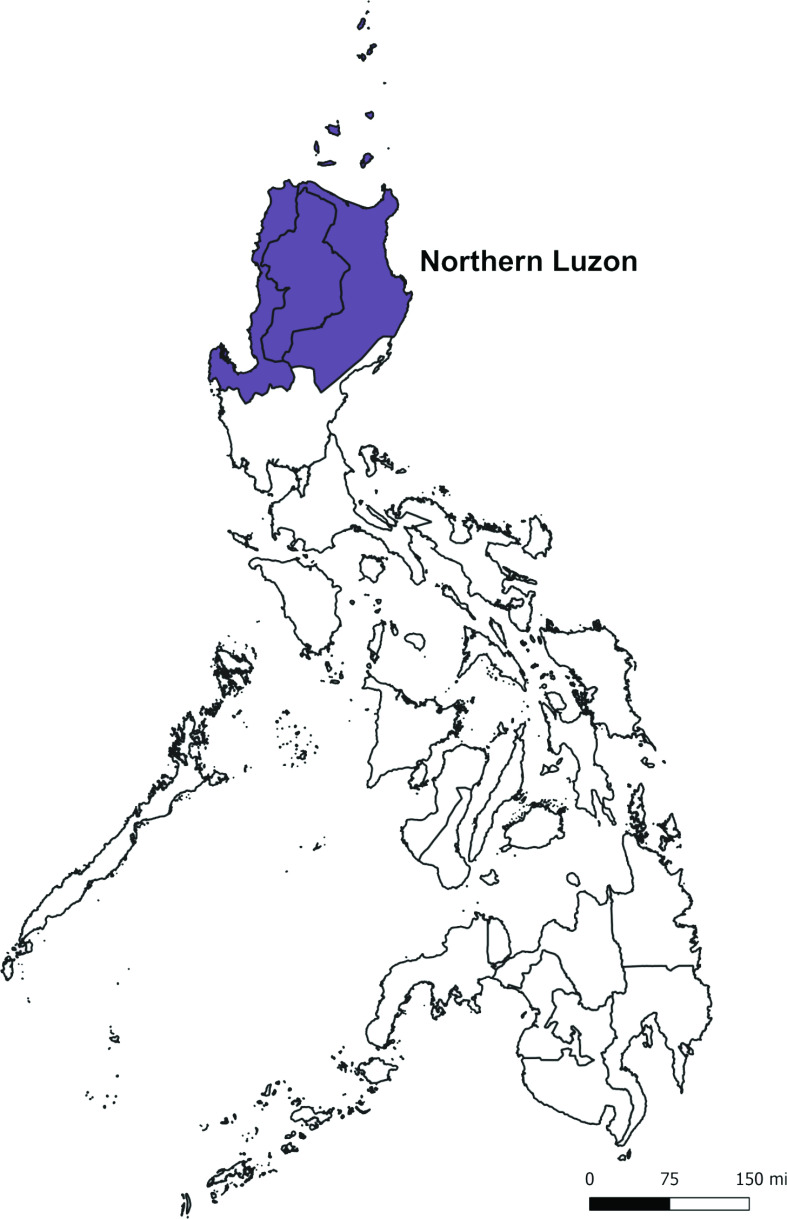
Map indicating Northern Luzon provinces, Philippines

A subnational initiative was implemented in Northern Luzon (**Fig. 1**) to address the shortage of practicing field epidemiologists. The Joint External Evaluation of IHR (2005) set the standard of one field epidemiologist per 200 000 population, (*3*) which the Philippines has not met.

The FETP in the Philippines is hosted by the DOH CHD with support from partners such as the DOH Epidemiology Bureau, the World Health Organization, the Field Epidemiology Training Program Alumni Foundation, Inc. (FETPAFI) and the South Asia Field Epidemiology and Technology Network (SAFETYNET).

[**insert** Figure 1]

## ACTION

The FETP–Intermediate Course (IC) aims to:

enhance the competencies of public health workers in data collection, analysis, interpretation and communication to support effective decision-making;
build the capacity at the subnational level to respond to outbreaks and other public health threats; and
foster a network of skilled field epidemiologists with a shared sense of purpose, working to common standards.

### Curriculum and competency domain

The FETP–IC curriculum is a 9-month modular training course that is based mainly on the CDC module and has been adapted to the Philippine context. It consists of five 1–2-week lecture series that provide participants with the knowledge and tools necessary to conduct job-relevant field projects. Individual projects are the centrepiece of the FETP–IC, requiring trainees to design and develop protocols, conduct field data collection, manage data, analyse data, interpret data, write reports and deliver oral presentations. Other on-the-job field projects include summarizing surveillance data, evaluating and making recommendations to improve a surveillance system, and investigating outbreaks. The field intervals between lecture series are 5–6 weeks, and participants are expected to complete field assignments while performing their usual job responsibilities.

Upon completion of the programme, participants should be able to summarize, identify and describe trends and patterns; interpret data from a surveillance system; conduct an outbreak investigation using descriptive epidemiology; design, conduct, analyse and interpret data from a descriptive epidemiological study; produce epidemiological reports; write an abstract; and deliver an oral presentation.

### Recruitment

Once the decision was taken to implement the FETP–IC, the recruitment and selection of trainees commenced. The first step involved interested applicants and local government units submitting a letter of intent to participate in the programme. After receiving the letters, the second step was a panel interview. The panellists were selected from FETP–Advance Course graduates, ensuring that the interviewers had the necessary experience and knowledge to determine the best candidates for the programme. A structured rating tool was used to maintain fairness and objectivity. Selection criteria were adapted from FETP Philippines to guide the panellists’ decision-making process.

The programme emphasizes the importance of teamwork among trainees, and participants are required to sign contracts with their local government units and the DOH to serve as epidemiologists after completing the programme.

### Master Trainer and mentors

In the programme, each cohort is assigned a Master Trainer, an experienced professional who has completed the FETP-Advance Course and is responsible for overseeing the progress of the trainees in their cohort. The Master Trainer provides guidance, feedback and support to the trainees throughout the duration of the programme.

In addition to the Master Trainer, coaching and mentoring are provided to the trainees by FETP graduates. These mentors are professionals who have successfully completed the FETP and have been trained to provide support and guidance to new trainees. Each mentor is responsible for supporting and coaching two trainees.

Through this coaching and mentoring system, the trainees receive personalized support and guidance as they go through the programme. The mentors provide feedback on the trainees’ performance, help them identify areas for improvement and provide guidance on how to address challenges that may arise during the programme. This system ensures that the trainees receive the necessary support and guidance to succeed in the programme and develop the skills needed to become effective epidemiologists.

### Training coordinator and staff

A dedicated training coordinator and training staff are responsible for ensuring the effective implementation of the training programme. They play a critical role in managing the logistics and administrative tasks associated with the programme, such as scheduling and organizing training sessions, coordinating with trainers and mentors, preparing training materials and tracking trainee progress.

### Sustainability

The programme’s graduates are awarded the title of Certified Applied Epidemiologist (CAE) through a CHD Regional Order, and the lecture series has been accredited by the Philippine Professional Regulation Commission, providing a maximum of 135 continuing professional development units. Moreover, a memorandum of agreement has been signed between the graduates’ hospital or local government unit and the CHD, signifying their commitment to collaborate continuously in enhancing epidemiology and surveillance in their respective areas.

## OUTCOME

### Establishment

In 2021, the DOH CHD of Cagayan Valley launched the first FETP–IC in the country, with 12 graduates deployed in the regional and local health offices of Cagayan Valley. In 2022, the CHD conducted their second batch with 12 trainees who graduated in March 2023.

On 16 May 2022, the DOH CHD of Cordillera Administrative Region, in partnership with Region I, launched the FETP–IC Northern Luzon Cluster with 18 trainees. This group comprised six trainees from the CHD, six from the provincial health offices, six from the municipal and city health offices, and two from hospitals.

The training team consisted of FETP–Advance Course graduates in partnership with SAFETYNET and FETPAFI.

### Accomplishments

Within 3 years, the two FETP–IC subnational training programmes produced 42 applied epidemiologists. As of February 2023, 92 studies had been conducted by the FETP–IC trainees, including 39 outbreak investigations, 37 data quality analysis/process improvement projects, 10 epidemiological studies and six surveillance evaluations. One of these studies has been published, (*4*) while three more are undergoing the peer review process. Six papers were accepted for presentation at the Bi-regional TEPHINET Scientific Conference to be held in September 2023, and another was accepted for presentation at the European Scientific Conference on Applied Infectious Disease Epidemiology in November 2023.

Trainees also conducted 37 trainings with a total of 700 participants in basic epidemiology, data management and analysis, disease surveillance, and the establishment of epidemiology and surveillance units (**Table 1**).

**Table 1:** Type and number of accomplishments of FETP–IC trainees, 2021–2023, Philippines

Accomplishment	No.
Outbreak investigations	39
Data quality analysis/process improvement projects	37
Epidemiological studies	10
Surveillance system evaluations	6
Case investigations	4
Trainings conducted by trainees	37^a^

FETP–IC: Field Epidemiology Training Programme–Intermediate Course.

^a^ Total of 700 participants.

### Notable studies conducted by trainees

A 2022 outbreak investigation in the municipality of Buiguias, Mountain Province, Philippines, found that only 15 out of 220 suspected cases of typhoid fever reported to the surveillance system corresponded to the case definition used in this study, indicating that the majority of reported cases did not meet the case definition. (*5*) The study highlighted the importance of improved surveillance and response systems for infectious diseases, as well as the need for control and preventive measures, including safe water and food handling practices, to prevent the spread of the disease in the municipality.

A 2021 study in Isabela on the mental health status of health-care workers involved in the COVID-19 response found that they faced stigma related to COVID-19, which affected their psychological well-being (unpublished). Health-care workers have reported symptoms of depression, anxiety and insomnia. The study recommended that policies be implemented to support mental health programmes at all levels and that changes be instituted, especially at the facility level, to address the growing problem of mental illness among health-care workers.

A 2021 evaluation of the dengue surveillance system in Cagayan Valley Region identified several challenges in terms of data quality, timeliness and sensitivity (unpublished). The study found that data quality needed improvement at all levels, as did timeliness and sensitivity ratings. The majority of cases were identified in hospitals, with most being dengue with warning signs. Although the study focused on a specific region, the challenges highlighted are not unique to this area.

A 2022 case study from a regional hospital showed the significance of effective surveillance and reporting systems for timely and accurate health data (unpublished). Consequently, the hospital made changes to improve their reporting, such as by creating an automated weekly surveillance report and by training link nurses. In 2022, the hospital achieved 100% reporting through the weekly surveillance reports from morbidity weeks 24 to 52. They also created a monitoring sheet, provided a laptop for surveillance activities, and reorganized their Hospital Epidemiology and Surveillance Unit.

## Discussion

The FETP–IC in the Philippines is an important effort to improve the country’s public health response capacity by building a skilled epidemiological workforce. The programme has produced 42 graduates from its first two subnational training programmes who will be deployed to strengthen epidemiology and surveillance in their respective localities.

Although not a formal evaluation of the FETP–IC, the outcomes reported in this study present convincing evidence of the immediate impact of the training. These outcomes demonstrate how the participants have used their newly acquired skills in data collection, analysis, interpretation and communication. Moreover, the training has aided effective decision-making at the subnational level, building the capacity to respond to outbreaks and address other public health hazards.

The programme’s impact can be seen in the number and variety of studies conducted by the trainees, including outbreak investigations, data quality analysis and process improvement projects, epidemiological studies and surveillance evaluations. These studies have helped to identify and respond to public health threats and have contributed to a better understanding of disease patterns and risk factors in the country.

Overall, the FETP–IC is an important investment in the country’s public health infrastructure. (*6*) By training and deploying skilled epidemiologists to local health offices and hospitals, the programme is helping to improve the capacity of the health system to respond to public health threats and protect the health of the population. The programme’s emphasis on practical training and real-world experience, as evidenced by the variety of studies conducted by trainees, is an effective way to build a strong and sustainable epidemiological workforce.
